# Large‐scale analysis of the genome of the rare alkaline‐halophilic *Stachybotrys microspora* reveals 46 cellulase genes

**DOI:** 10.1002/2211-5463.13573

**Published:** 2023-02-17

**Authors:** Salma Abdeljalil, Ines Borgi, Ines Ben Hmad, Fakher Frikha, Olivier Verlaine, Bilal Kerouaz, Nesrine Kchaou, Ali Ladjama, Ali Gargouri

**Affiliations:** ^1^ Molecular Biotechnology of Eukaryotes Laboratory, Centre of Biotechnology of Sfax University of Sfax Tunisia; ^2^ Laboratory of Molecular and Cellular Screening Processes, Center of Biotechnology of Sfax University of Sfax Tunisia; ^3^ Bacterial Physiology and Genetic Institute, Centre for Protein Engineering University of Liège Belgium; ^4^ Laboratory of Applied Biochemistry and Microbiology, Department of Biochemistry, Faculty of Sciences University Badji Mokhtar Annaba Algeria; ^5^ Analytical Services Unit at the Center of Biotechnology of Sfax Tunisia

**Keywords:** cellulases, functional annotation, halophilic profile, MiSeq sequencing, *Stachybotrys microspora*

## Abstract

Fungi are of great importance in biotechnology, for example in the production of enzymes and metabolites. The main goal of this study was to obtain a high‐coverage draft of the *Stachybotrys microspora* genome and to annotate and analyze the genome sequence data. The rare fungus *S. microspora* N1 strain is distinguished by its ability to grow in an alkaline halophilic environment and to efficiently secrete cellulolytic enzymes. Here we report the draft genome sequence composed of 3715 contigs, a genome size of 35 343 854 bp, with a GC content of 53.31% and a coverage around 20.5×. The identification of cellulolytic genes and of their corresponding functions was carried out through analysis and annotation of the whole genome sequence. Forty‐six cellulases were identified using the fungicompanion bioinformatic tool. Interestingly, an *S. microspora* endoglucanase selected from those with a low isoelectric point was predicted to have a halophilic profile and share significant homology with a well‐known bacterial halophilic cellulase. These results confirm previous biochemical studies revealing a halophilic character, which is a very rare feature among fungal cellulases. All these properties suggest that cellulases of *S. microspora* may have potential for use in the biofuel, textile, and detergent industries.

## Graphical abstract

The goal of this study was to obtain a high‐coverage draft of the genome of the rare alkaline‐halophilic *Stachybotrys microspora* and to annotate the genome sequence. Using two bioinformatics tools, fungicompanion and OmicsBox, we identified several cellulase genes, suggesting this species may have potential for use in several industries.

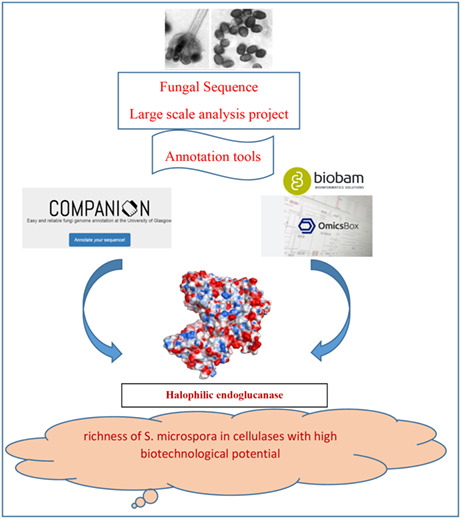

**Abbreviations**
BglG: β‐glucosidase G
CBM: carbohydrate‐binding module
EG: endoglucanase
KDa: kilo Dalton
NGS: next generation sequencing
PDB: Protein Data Bank

Since the completion of the first human genome sequence, the demand for cheaper and faster sequencing methods has increased dramatically. This demand has led to the development of second‐generation or next‐generation sequencing (NGS) sequencing methods. The NGS platform performs massively parallel sequencing (sequencing millions of DNA fragments from a single sample) that facilitates high‐throughput sequencing, allowing the entire genome to be sequenced in less than a day [1]. The creation of the NGS platform allows laboratories to access sequences and rapidly increases the number of research results in multiple fields such as fungal biology research.

Fungi have immeasurable impacts on ecosystems, and are so important in ecology, agriculture, medicine, and biotechnology that they affect many aspects of society. They are used as suitable organisms for basic research because of the typical haplobion life cycle that facilitates the study of the phenotype of mutations and the ability of most cells to differentiate throughout the organism. In addition, many fungi are easy to culture and are useful for microbiological, genetic, and molecular techniques. Therefore, fungi were one of the first model organisms for the study of genetics, biochemistry, cells, and developmental biology. Therefore, it is not surprising that the first eukaryote for which the complete genomic sequence was obtained was the fungus *Saccharomyces cerevisiae* [1].

Fungi are also very important in biotechnology and in the manufacture of drugs and enzymes. The first antibiotic Alexander Fleming isolated was derived from a fungal strain of *Penicillium* species. Today, the genomes of over 1000 filamentous fungi have been sequenced. NGS technology has revolutionized genomic resequencing. In strain comparisons, gene mapping, or transcriptome and ChIP analysis, the *de novo* assembly of the eukaryotic genome still represents a significant hurdle due to its size and range of repeats [2]. Filamentous fungi are good candidates because the 30–90 Mb genome contains few repetitive regions and is expected to assemble from short sequence reads much easier than mammals and higher plants [3]. We are working on a rare, locally isolated fungal strain belonging to the *Stachybotrys microspora*. *Stachybotrys* is related to the genus *Memnoniella*, most *Stachybotry*s species inhabit cellulose‐rich substances [4]. This genus is widely distributed and contains about 50 species [5]. Interestingly, our strain is characterized by growing in a cellulosic‐based medium over a wide pH range of 4–9. This is rarely reported in most known fungal species. In addition to its ability to grow at alkaline pH, *S. microspora* produces neutral and alkaline endoglucanases [6] and secretes several β‐glucosidases [7, 8, 9, 10, 11, 12, 13], whereas fungi generally have one or two β‐glucosidase. It has been shown that at least six β‐glucosidases are produced experimentally [7, 8, 9, 10, 11, 12, 13], but the number of genes is probably much higher (see below in the Results section). One of these β‐glucosidases, called BglG, although produced constitutively on 1% glucose, is inhibited by glucose, retaining only 33% at 10 mm glucose while stimulated to 120% by the same xylose concentration [14]. In this regard, it should be noted that one of the major challenges in the bioconversion of lignocellulosic biomass to liquid biofuels involves the search for sugar‐tolerant β‐glucosidases, as glucose inhibits β‐glucosidase activities. β‐Glucosidase is one of the key enzymatic components of cellulase that converts cellobiose into glucose to complete the final stages of cellulose hydrolysis. This removes the inhibition exerted by cellobiose on cellobiohydrolase, the enzyme that initiates the attack on cellulosic substances. Therefore, β‐glucosidase plays a very important role in the enzymatic production of bioethanol from biomass [14, 15, 16, 17].

To take advantage of the potential of this strain, we conducted a molecular study of the cellulolytic genes to investigate the molecular expression profile of the corresponding gene. We succeeded in isolating three β‐glucosidase genes from the glycoside hydrolases 1 and 3 families, confirming the presence of β‐glucosidase, and showed different expression patterns [9, 10]. In addition to secreting cellulase, the fungus produces xylanase, protease, chitinase, β‐glucanase, and pectinase, some of which share the same regulatory mechanism to induce or suppress their respective activities [18]. In addition to its richness in β‐glucosidases, our fungus is distinguished by the very specific properties of its endoglucanases. Indeed, we have shown that it secretes two enzymes, called EG1 and EG2, which are very interesting for their high salt tolerance [19, 20]. Indeed, EG1 is the most halophilic fungal endoglucanase ever studied, with an optimal activity at 5.6 m NaCl and resists SDS by retaining more than 70% of its activity at 10% SDS [19]. Moreover, this enzyme is active in the presence of ion liquids [20].

For all these interesting data, sequencing the entire genome of this rare fungus is crucial for advancing knowledge of its genomic properties. We analyzed the genome sequence of this strain and identified 46 cellulase genes. The halophilic profile was studied through the physicochemical parameters of one endoglucanase predicted to be halophile and through its molecular modeling.

## Materials and methods

### Extraction of genomic DNA

*Stachybotrys microspora* was grown on potato dextrose agar medium at 30 °C for 4–7 days. Spores were harvested in 0.1% Tween 80 solution and used to inoculate modified Mandels' medium [21], per liter: 2 g KH_2_PO_4_, 1.4 g (NH_4_)_2_SO_4_, 1 g yeast extract, 0.69 g urea, 0.3 g CaCl_2_·2H_2_O, 0.3 g MgSO_4_·7H_2_O, 1 mL Tween 80, and 1 mL trace element solution composed of 1.6 g·L^−1^ MnSO_4_, 2 g·L^−1^ ZnSO_4_, 0.5 g·L^−1^ CuSO_4_, and 0.5 g·L^−1^ CoSO_4_.

Cells were cultivated for 5 days at 30 °C in shaking flasks containing 100 mL of Mandels' medium in Erlenmeyer's flasks (500 mL), supplemented with 100 μg·mL^−1^ ampicillin and 12 μg·mL^−1^ tetracycline to avoid bacterial contamination, and shaken at 150 r.p.m. rotary agitation.

Genomic DNA was extracted as follows: 1 g of frozen mycelium was ground into fine powder with alumina and mixed with 5 mL of extraction buffer [10 mm Tris–HCl (pH 8.0), 50 mm EDTA and 0.5% SDS]. After two extractions with an equal volume of phenol equilibrated with Tris–HCl, the DNA was precipitated overnight with 0.1 times the amount of 3 m sodium acetate (pH 5.2) and 2.5 times the amount of ethanol [22]. DNA quality and concentration were determined by horizontal gel electrophoresis on a 0.8% agarose gel in 50 mm Tris‐Acetate‐EDTA buffer, pH 8.

### Illumina MiSeq analysis and assembly

Sequencing libraries were prepared using the Nextera XT DNA Library Prep Kit (Illumina, San Diago, CA, USA) and genomic sequencing was performed using the MiSeq Sequencer (Illumina) and Reagent Kit v3 (Illumina). Unless otherwise stated, default values were used throughout the software.

Run duration is based on the number of cycles performed. We performed a paired‐end run up to 2 × 301 sequencing cycles plus any Index Reads with mcs v2.3. *De novo* assembly of the raw data was performed using spades v3.11.1 [23]. The assembly uses the default settings and adds an option to modify the reading.

N50: statistic defines assembly quality in terms of contiguity. Given a set of contigs, the N50 is defined as the sequence length of the shortest contig at 50% of the total assembly length. It can be thought of as the point of half of the mass of the distribution; the number of bases from all contigs longer than the N50 will be close to the number of bases from all contigs shorter than the N50.
N50 can be described as a weighted median statistic such that 50% of the entire assembly is contained in contigs or scaffolds equal to or larger than this value.
L50: Given a set of contigs, each with its own length, the L50 is defined as the count of the smallest number of contigs whose length sum makes up half of the genome size.

### Bioinformatics tools for functional annotation

Functional annotation and analysis were performed using OmicsBox (available at https://www.biobam.com/) and the fungicompanion web server (http://fungicompanion.gla.ac.uk/).

### Screening for fungal cellulases

Cazy Carbohydrate Active Enzymes (http://www.cazy.org/), dbCAN Metaserver for automated CAZyme annotations: https://bcb.unl.edu/dbCAN2/ and Fungal Genomics Resource «MycoCosm»: https://mycocosm.jgi.doe.gov/mycocosm/home, were used to compare cellulases between *S. microspora* and the most related fungi.

### Phylogenetic analysis of marine fungi

In order to investigate the halophilic feature of produced cellulases from *S. microspora*, the entire 18S ribosomal sequences from marine fungi were aligned using the multiple sequence alignment tool of the muscle algorithm, integrated in the mega x package, and subjected to cluster analysis by the distance with the neighbor‐joining method using mega x software (Molecular Evolutionary Genetics Analysis, https://www.megasoftware.net/).

### Homology modeling and docking

The secondary structure of the protein sequence deduced from the studied *S. microspora* endoglucanase was modeled by homology with a Swiss‐model server [24] using the structure of a tri‐modular halophilic bacterial cellulase with a family 46 carbohydrate binding module (PDB: 5e09.1.A) as a template [25]. The built model was displayed in the software swiss pdb viewer v4.01 [26]. A ProSA server was used to evaluate the parameters and predict the quality of the modeled structure [27].

The Molecular Operating Environment MOE 2019 (moe) software was used for molecular mechanics optimization, Protein Property Descriptors, and structure visualization. Within moe, the 3D structure of the halophilic and nonhalophilic endoglucanase was repaired for missing atoms, charges were assigned according to the OPLS‐AA force field, and molecular mechanics optimization until the gradient of 0.01 kcal·(Å mol)^−1^ was reached. Its properties were then calculated across a range of pH values pH = [4, 10] and ionic strengths ‘NaCl’ *I* = 0.1 m with temperature *T* = 310 K, Viscosity η = 0.89 (cP), and Dielectric ε_*r*_ = 78.

protparam (https://web.expasy.org/protparam/) was used as a tool to enable the calculation of various physical and chemical parameters of the predicted GH5 endoglucanase. Calculated parameters include molecular weight, theoretical pI, amino acid composition, atomic composition, extinction coefficient, estimated half‐life, instability index, aliphatic index, and overall mean hydropathy (GRAVY).

Molecular docking was performed using the SwissDock server (http://www.swissdock.ch/). SwissDock is based on the docking software eadock dss, whose algorithm consists of many steps [28, 29]. The target molecule was provided as a PDB file. Binding mode was evaluated and clustered using FullFitness. The clusters were then ranked based on the perfect fit of those elements. In subsequent cycles, the structure with the lowest “Full Fitness” and the estimated Δ*G* value was selected and the adjacent docked ligand structures were collected as representatives. In other words, of the many binding modes generated, only the minimal energy conformational state of the ligand‐binding protein complex was considered. Binding mode results were visualized and analyzed by Swissdock using ucsf chimera 1.14 [30].

## Results and Discussion

### Genome sequencing and assembly

The *S. microspora* genome was sequenced using the next‐generation Illumina‐MiSeq sequencer, and genome assembly was performed using the spades program.

The raw data included 5 374 208 paired reads, with an exact length of 301 bp, that were used for assembly. Gene prediction was performed using augustus v2.0 [31], and the final annotation included 10 830 protein‐coding genes. The genome sequence had 35 343 854 bp, with a GC content of 53.31% and a 20.5‐fold coverage. The total number of contigs was 3715, the values of N50, N75, L50, and L75 were 15.961, 7.477, 644, 1463, respectively.

### Functional annotation with the Omicsbox platform

**Fig. 1:**
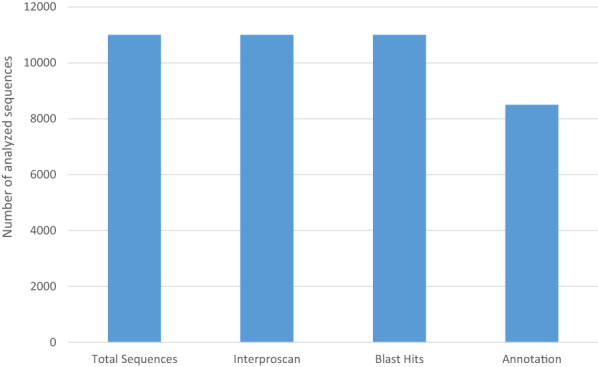
Analysis process of the Omicsbox platform of a total of 11 000 sequences. InterPro protein signatures and domain hits were obtained using interproscan. The mapping step links the different protein IDs to the functional information of the Gene Ontology database followed by the annotation process.

blast hits were imported into OmicsBox to perform Gene Ontology mapping and annotation. InterPro protein signatures and domain hits were obtained using interproscan. The output was then imported into OmicsBox and merged with the GO annotation and mapping results (Fig. 1).

In fact, the full Gene Ontology annotation workflow used cloud‐powered algorithms (blast, interproscan, go mapping) to get the most complete annotation labels. Detailed statistics of every step were generated to summarize the results. Combined graphs for the three categories (Biological Process, Molecular Function, and Cellular Component) were generated including the annotations from interproscan results. To give an idea regarding the OmicsBox workflow, an example of the output after performing annotation and a sample results analysis of family 3 β‐glucosidase (Figs [Link], [Link]).

**Fig. 2:**
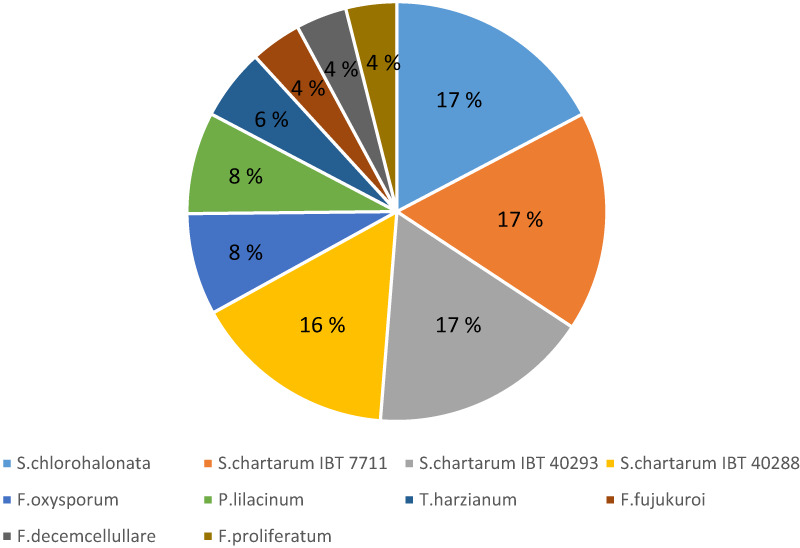
Species distribution of the highest blast hits under the Omicsbox platform. The percentage of homology for each organism in indicated in the pie chart.

A total of 11 000 sequences included in a fasta file as an input were blasted and only 8500 proteins were annotated with a given function. Moreover, the analysis of species distribution for the highest blast hits indicated a high homology with the closely related fungi *Stachybotrys chlorohalonata* and *Stachybotrys chartarum* (approximately 100% similarity for some cellulases genes like GH1 β‐glucosidases and one GH5 endoglucanase) but also with many species from the genus *Fusarium* (Fig. 2).

**Fig. 3:**
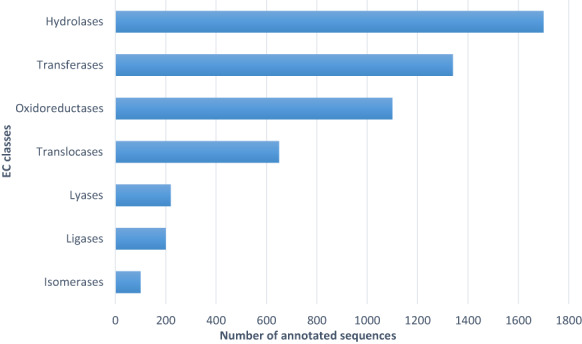
Distribution of enzymes classes: oxidoreductases, transferases, hydrolases, lyases, isomerases, ligases, and translocases. One thousand and seven hundred sequences were annotated to be hydrolases.

The study of the enzyme distribution in the Omicsbox platform gave that most of the annotated proteins are enzymes belonging to hydrolases, transferases, oxidoreductases, translocases, ligases, lyases, and isomerases (Fig. 3), with a high level of hydrolases in comparison with other classes of enzymes (Fig. 3). In fact, hydrolases englobe classical enzymes such as proteases and lipases but also glycoside hydrolases, a widely distributed group of carbohydrate active enzymes (CAZy) that hydrolyze glycosidic bonds into glycosides, glycans, and glycol‐conjugates [32].

### Functional annotation with the fungicompanion web server

To address the demand for quick, automatically generated genome annotations, we used the fungicompanion web server (http://fungicompanion.gla.ac.uk/). This server allows uploading the target assemblies and select a closely related reference species to guide the annotation.

The resulting annotation using the fungicompanion web server is summarized in Table 1. Unfortunately, the fungicompanion server has its own limitations in the choice of the reference genome. In the absence of *S. chlorohalonata* and *S. chartarum* in the server, we were forced to use only *Aspergillus fumigatus* and *Fusarium graminarium*, as they are the closest species to our strain among all the options offered by the program.

**Table 1:** The results of annotation analyses with the fungicompanion web server using the genome of *Aspergillus fumigatus* as a reference.

Analysis	Value
Number of annotated regions/sequences	5
Gene density (genes/megabase)	142.33
Number of genes	4093
Number of coding genes	4003
Number of noncoding genes	90
Number of genes with function	3273
Number of genes with multiple CDS	2489
Overall GC%	52.76
Coding GC%	56.76

Moreover, we were also forced to reduce the total number of nodes, as the annotation under this server is limited to 3000 nodes. A comparative table regarding the annotation of cellulase genes between the Omicsbox platform and the fungicompanion web server (Table 2) indicates some differences between these tools precisely in the number of predicted proteins, which is higher with Omicsbox (around 10 830) than with the fungicompanion web server (5225 and 5931 using as references the genomes of *A. fumigatus* and *F. graminarium*, respectively).

**Table 2:** Comparative functional annotation between the OmicsBox platform (*Stachybotrys microspora de novo* sequencing) and the fungicompanion web server (*Aspergillus fumigatus* and *Fusarium graminearum* genomes as references) regarding cellulolytic enzymes.

Tool of annotation	Fungicompanion online server		OmicsBox 1.4.11 (Biobam)
Reference genome	*A. fumigatus*	*F. graminearum*	*S. microspora de novo* sequencing
Genome size (Mbp)	30	37	35
Total proteins (CDS)	5225	5931	10 830
β‐Glucosidases	18	17	16
Endoglucanases	22	27	17
Exoglucanases	4	3	10
Total cellulases	44	47	43

### Screening of cellulases genes

The predicted cellulases (β‐glucosidases, exoglucanases, and endoglucanases), using fungicompanion and the Omicsbox platform, are presented in Tables 2 and 3, with a total of 46 cellulases for the fungicompanion and 43 for the Omicsbox platform. Moreover, the annotation of the *S. microspora* genome using the dbCAN meta server with Diamond, Hmmer, and Hotpep programs gave comparable results (data not shown).

**Table 3:** Comparative cellulases secretion among *Stachybotrys microspora*, *Stachybotrys chlorohalonata*, *Stachybotrys chartarum*, *F. oxysporium*, and *Trichoderma harzianum* using Fungicompanion program.

Fungus	β‐Glucosidase (GH1 and GH3)		(Endoglucanases)	Cellobiohydrolases (GH6 and GH7)		Total
*S. chlorohalonata*	2	20	18	5	7	52
*S. chartarum*	2	25	17	5	6	55
*F. oxysporium*	6	27	21	1	3	58
*T. reesei*	7		6	2		15
*T. harzianum*	2	16	12	1	2	33
***S. microspora***	**2**	**16**	**18**	**10**		**46**

An inventory of cellulases enzymes was performed to compare the richness of the GH families between *S. microspora* and the most related fungi according to the genomic analyses: *Fusarium oxysporum*, *S. chlorohalonata*, *S. chartarum*, and *Trichoderma harzianum* (Table 3). The results show that *Fusarium oxysporium* possesses more cellulase enzymes than the other studied fungi and shares the same number of endoglucanases with *S. microspora*. However, a higher number of cellobiohydrolases are found in *S. chlorohalonata* and *S. chartarum*.

This table shows that *T. harzianum* and *S. microspora* share the same high number of β‐glucosidases, which it is not the case for *Trichoderma reesei*, which shows only 7 β‐glucosidases from the GH3 glycoside family [33]. This result confirms the richness of our strain concerning the production of β‐glucosidases and endoglucanases (only two cellobiohydrolases and six endoglucanases for *T. reesei* against 10 cellobiohydrolases and 17 or 18 endoglucanases (depending on the platform used) secreted by *S. microspora*), which is considered a very interesting characteristic for further industrial use.

It should be noted that the number of the predicted endoglucanases and β‐glucosidases differ, depending on the annotation tool used, but the predicted exoglucanases (cellobiohydrolases) number is the same whether it was predicted using the fungicompanion tool or the Omicsbox platform.

### Phylogenetic analysis

Biochemical studies on *S. microspora* have shown a halophilic character of secreted endoglucanases [19, 20]. Moreover, some strains form *Stachybotrys* genera are considered a marine fungus like *S. chartarum* according to the World Register of Marine Species (https://fr.wikipedia.org/wiki/Stachybotrys#WRMS), *S. longispora* [33], and *Stachybotrys* sp. Strain MF347 [34]. In fact, having a marine origin is concordant with somehow the halophilic feature for some fungal secreted enzymes. Therefore, we performed a multiple alignment of 18S ribosomal sequences (Fig. S4) and constructed a phylogenetic tree for some known marine fungi with *S. chartarum* and *S. chlorohalonata* compared to *S. microspora* (Fig. S4). We note that the most related fungi to *S. microspora* is *S. chartarum*, sharing the same clade in the tree.

In this context, the purification of the EG1 led us to reflect on the halophilic character of our strain, since this enzyme has the strongest resistance to NaCl ever described for a cellulase, i.e., 5.6 m [20]. In fact, it must be said that although EG1 served as the instigator of our research, this protein has not yet been identified as a protein sequence or as a gene since we have not yet succeeded in determining its N‐terminal sequence, despite several attempts. We think it would be blocked and we will try to use the LC–MS/MS to approach it. All we know about EG1 is its size, 55 kDa, its extreme resistance to high concentrations of NaCl, and its other biochemical properties reported in our article [20]. In the meantime, we tried in this study to see if there were candidate sequences with halophilic profiles. So, for now, the model is chosen only on the basis of size and halophilia.

The analysis of known halophilic enzymes has shown that such enzymes are endowed with an isoelectric point pI value between 4 and 5 and a higher number of acidic amino acids residues compared to other basic ones [35]. More important, the structure prediction of such enzymes indicates that the acidic amino acids (negatively charged amino acids) are predominantly present on the protein surface, giving a tertiary structure entirely colored in red [36]. In this context, we compiled data on annotated *S. microspora* endoglucanases regarding their pI, size, and ratio of negatively charged versus positively charged amino acids (Table 4).

**Table 4:** Analysis of all annotated endoglucanases from *Stachybotrys microspora* regarding physicochemical properties. The sequence seq4 and seq10 correspond to the nonhalophilic and the halophilic endoglucanases shown in Fig. 4. In bold: all the endoglucanases with a low pI.

Endoglucanase	1	2	3	4	5	6	7	8	9	**10**	11	12	13	14	15	16	17
Molecular weight (kDa)	56	35	25	44	36	32	41	26	37	**59**	68	46	46	24	27	33	26
pI	6	4	4.4	6	4	6.4	7.7	8.9	5.1	**4.2**	5.5	4.3	4.3	4.5	4.4	5.6	4.3
Positively charged amino acids	57	8	12	28	13	21	27	13	21	**26**	44	23	21	9	12	15	12
Negatively charged amino acids	69	32	23	32	48	24	26	10	32	**73**	52	56	53	21	29	19	30

Table 4 reveals that seq2, 10, 12, 13 and many others show the same range of pI (around 4 and 4.5) and electronegativity; but we chose the seq10 for the following reasons. (a) seq10 showed the highest value of total solvent accessibility around 23 590 compared to seq12 (13 842.7) and seq13 (16 620.3); (b) the area of protein patches negative for seq10 is higher than those for seq12 and seq13 (Table 6). That said, seq13 is closer to seq10 and *Bacillus* sp. *BG* as seq12; (c) we did not analyze seq2 and other endoglucanases (having pI in the same range as seq10) due to their small sizes, considering the large size of EG1.

**Fig. 4:**
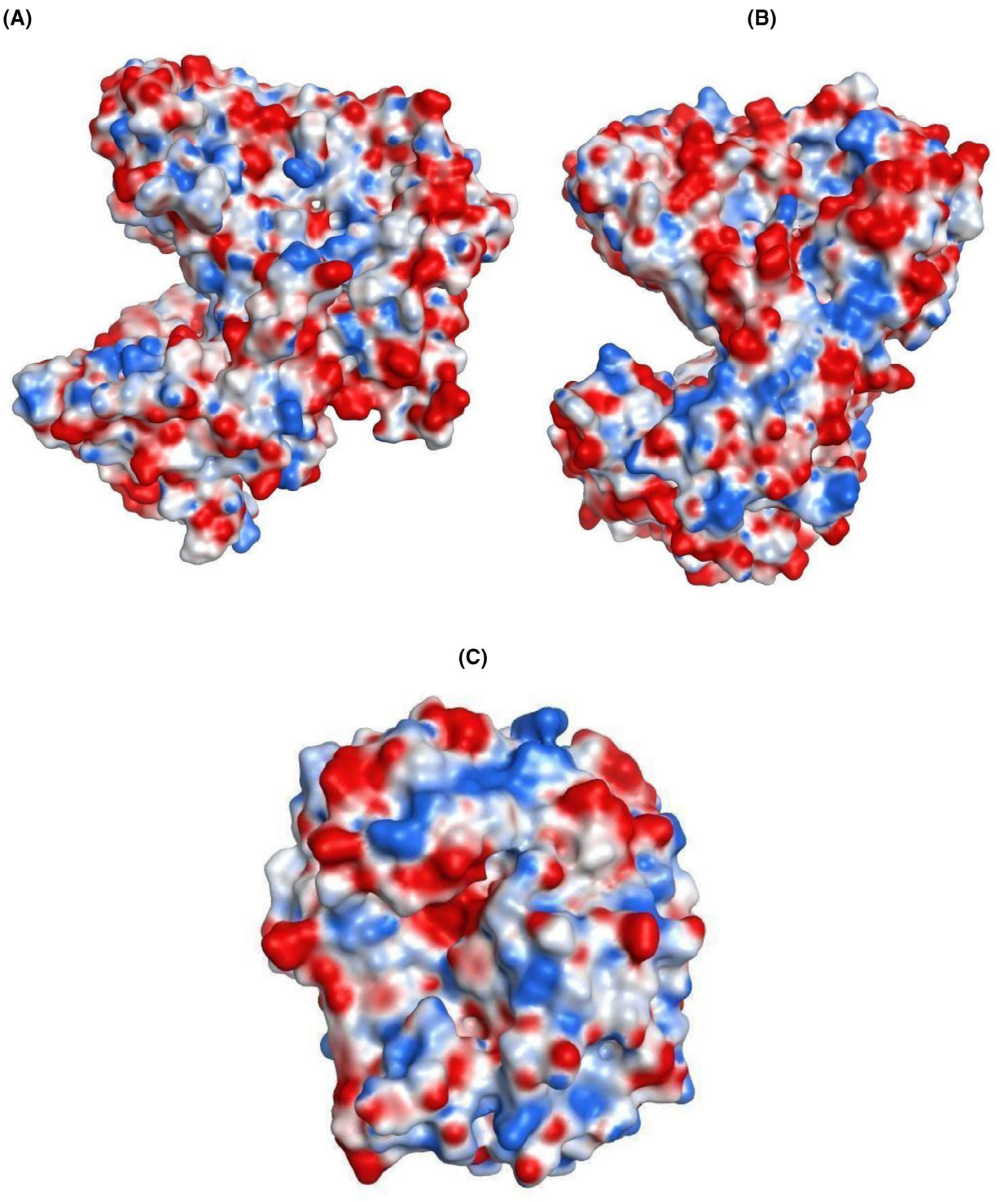
Electrostatics molecular surface of nonhalophilic and halophilic endoglucanase. The electrostatic color map ranges from −40 (negative charged, red color), through 0 (noncharged, white color) to +40 (positive charged, blue color). (A) Visualization of the predicted trimodular model from *Stachybotrys microspora* GH5 endoglucanase with the Molecular Operating Environment MOE 2019 (moe) software showing acidic residues (red) and basic residues (blue). (B) Visualization of the template: (PDB: 5e09.1.A) from *Bacillus* sp. *BG* with the moe software showing acidic residues (red) and basic residues (blue). (C) Visualization of the predicted nonhalophilic endoglucanase from *S. microspora* with the moe software showing acidic residues (red) and basic residues (blue). Note that these nonhalophilic and the halophilic endoglucanases correspond to the sequence seq4 and seq10 in the Table 4, respectively.

Therefore, seq10 was chosen for further analyses. Indeed, seq10 is a GH5 endoglucanase that presented a molecular weight of 59 kDa and a theoretical pI of 4.23; its predicted physicochemical properties give a halophilic profile with a total number of negatively charged residues (Asp + Glu) of 73 and 26 positively charged residues (Arg + Lys). Moreover, the electrostatic potential analyses indicate that almost the entire solvent‐accessible surface of the studied endoglucanase is negatively charged, which is consistent with the halophilic nature of this enzyme (Fig. 4A). The instability index (II) was computed to be 31.76 and classifies the protein as stable.

### Homology modeling of the predicted halophilic endoglucanase

More interestingly, the seq10‐GH5 endoglucanase from *S. microspora* showed 33.60% sequence identity with a tri‐modular halophilic cellulase from *Bacillus* sp. BG displaying a Carbohydrate‐Binding Module from the Family 46 (CBM46), a typical (β/α)8 TIM barrel catalytic domain and a CBM_X domain (PDB: 5e09.1.A), as determined by Zhang *et al*. [25].

**Fig. 5:**
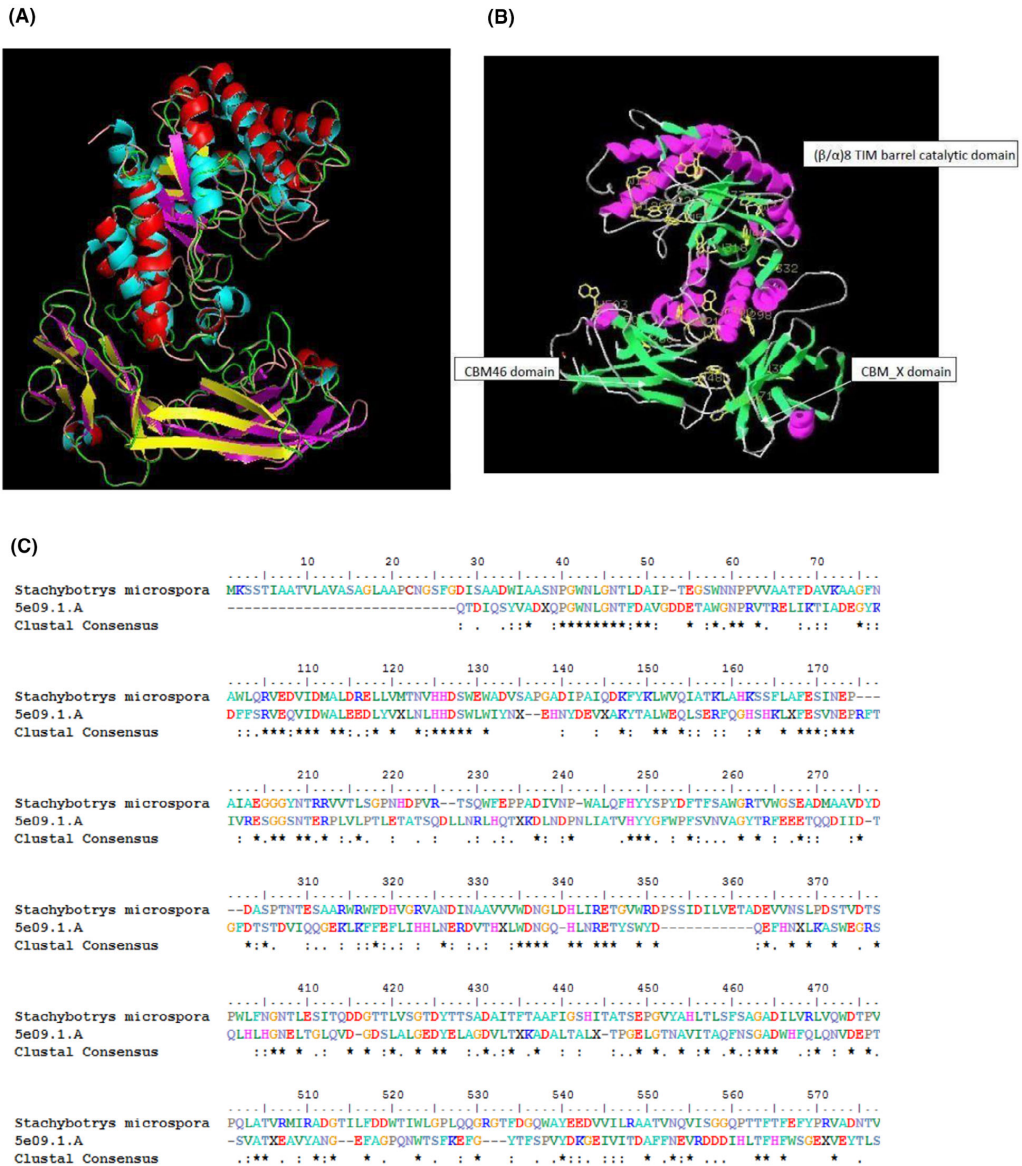
(A) Visualization of a superposition of *Stachybotrys microspora* endoglucanase (colored in red Helix, yellow β‐Sheet, and green for loop) with the published halophilic GH5 structure: 5e09.1.A (colored in blue Helix, β‐Sheet with magenta color and loop in pink). (B) Visualization of *S. microspora* model showing key amino acids: Tryptophane (colored in yellow) and the different domains: (β/α)8 TIM barrel catalytic domain, CBM46 domain, and CBM_X domain. (C) Multiple sequence alignment of *Stachybotrys microspora* GH5 halophilic endoglucanase (seq10 from Table 4) with the template PDB: 5e09.1.A (endoglucanase from *Bacillus* sp. *BG*).

The superposition (Fig. 5A) between this template (Fig. 4B) and the model (Fig. 4A) from *S. microspora* (Fig. 5B) presented a typical (β/α)8 TIM barrel catalytic domain, a CBM_X domain, and a CBM46 domain, reported previously [25]. Also, electrostatic potential analysis indicates that almost the entire solvent accessible surface of CelB is negatively charged [36].

A multiple sequence alignment of *S. microspora* GH5 halophilic endoglucanase (seq10 from Table 4) was performed using the template PDB: 5e09.1.A (endoglucanase from *Bacillus* sp. *BG*) showing some significant homology in different regions (Fig. 5C).

The parameters and prediction quality of the modeled structure were evaluated using the ProSA server [27]. The overall model quality gave a *Z* score of −7.65. The determined scores are within the range of native proteins of similar size (data not shown). The catalytic domain shows the features of the GH5 family, with a typical (β/α)8 TIM barrel, while the CBM46 domain has a sandwich‐like structure. The catalytic and the CBM46 domains form an extended substrate‐binding cleft, within which several tryptophan residues are well exposed. Such residues are proven to be important for substrate binding. Also, almost the entire surface of CelB is negatively charged, which is a signature of halophilic proteins [25].

Table 5 indicates also the differences regarding the physicochemical properties between the endoglucanase from *S. microspora*, the template from *Bacillus* sp. *BG* «5e09.1», and the predicted nonhalophile endoglucanase from *S. microspora*.

**Table 5:** Physicochemical properties of predicted halophile and nonhalophile endoglucanases from *Stachybotrys microspora*, *Bacillus* sp. *BG* « 5e09.1.A ».

Enzyme	Predicted halophile *S. microspora* (seq10 in Table 4)	*Bacillus* sp. *BG*	Predicted nonhalophile *S. microspora* (seq4 in Table 4)
Molecular weight (kDa)	59	65	44
pI	4.23	4.62	5.99
Positively charged amino acids	**26**	**36**	**28**
Negatively charged amino acids	**73**	**88**	**32**

We recall that proteins, and more generally solutes, are often charged in solution. Knowledge of their electro‐hydrodynamic properties is of utmost importance in the design of pharmacological formulations and systems within which these are to be manipulated. When these charged objects are suspended in a buffer solution of a given pH and ionic strength I, there is a cloud of ions that forms around them, which dampens the perceived electrostatic field and gives the object an apparent charge that will dictate how it reacts to external electrostatic fields [37].

The properties structure of the halophilic and nonhalophilic endoglucanase (Fig. 4C) was calculated by the Ensemble Protein Properties application in moe, which provides all calculations of properties and specialized descriptors for proteins with built‐in ensemble sampling and pH dependence as a central feature of the calculation. These properties were calculated across a range of pH values pH = [4, 10] and ionic strengths *I* = 0.1 m with *T* = 310 K, η = 0.00089 Pa·s, and ε_*r*_ = 78. The results are shown in Table 6.

**Table 6:** Description of physicochemical and structural studies properties of the predicted halophile (seq10), seq12, seq13 and the nonhalophile endoglucanase (seq4) from *Stachybotrys microspora*, and the template (reference: 5E09.1.A).

Description value at (pH = 7.00)	Endoglucanase nonhalophile seq4	Endoglucanase halophile seq10	*Bacillus* sp. *BG* 5E09	seq12	seq13
Area of hydrophobic protein patch(es)	996	2240	630	1192	1162
Area of positive protein patch(es)	603 (43%)	537 (16%)	250 (08%)	606 (37%)	565 (21%)
Area of negative protein patch(es)	812 (57%)	2779 (84%)	2930 (92%)	1024 (63%)	2151 (79%)
Area of ionic protein patch(es)	1415 (100%)	3316 (100%)	3180 (100%)	1629 (100%)	2716
Protein mass in kDa	34.38	58.98	60.88	39.37	43.39
Sequence‐based pI prediction	4.89	3.79	4.2	4.45	3.79
Structure‐based pI prediction	5.9	4.02	3.84	5.22	3.97
Radius of gyration	18.34	25.49	25.59	19.17	20.82
Hydrodynamic radius	25.63	33.66	32.51	27.13	28.96
Accessible surface area (water probe)	12 476.2	23 027.2	20 668.3	13 846.2	16 232.7
Hydrophobic surface area	5798.8	12 128.4	8948.1	7061.2	7677.7
Hydrophilic surface area	5732.5	8962.9	9566	5757.4	7171.7
Protein volume	31 707.1	54 424.6	55 736.1	36 209.9	39 457.6
Protein mobility	−6.1	−29	−41	−10	−29
Protein net charge	−5.45	−38.3	−49.82	−10.12	−30.72
Protein dipole moment	460.17	719.62	770.12	708.54	764.05
Hydrophobicity moment	466	972.1	1077.3	931.6	1040.2

At pH 7, the analysis of the structures surface of the three proteins (nonhalophilic, halophilic, and reference) share that the ratio (area of ionic protein patch(es)/accessible surface area) is almost similar (11%, 14%, and 15%, respectively). However, we note that the area of the negative protein patch is much higher for halophilic endoglucanase and the reference than the nonhalophilic endoglucanase (2779 and 2930 vs. 812 A^2^, respectively).

**Fig. 6:**
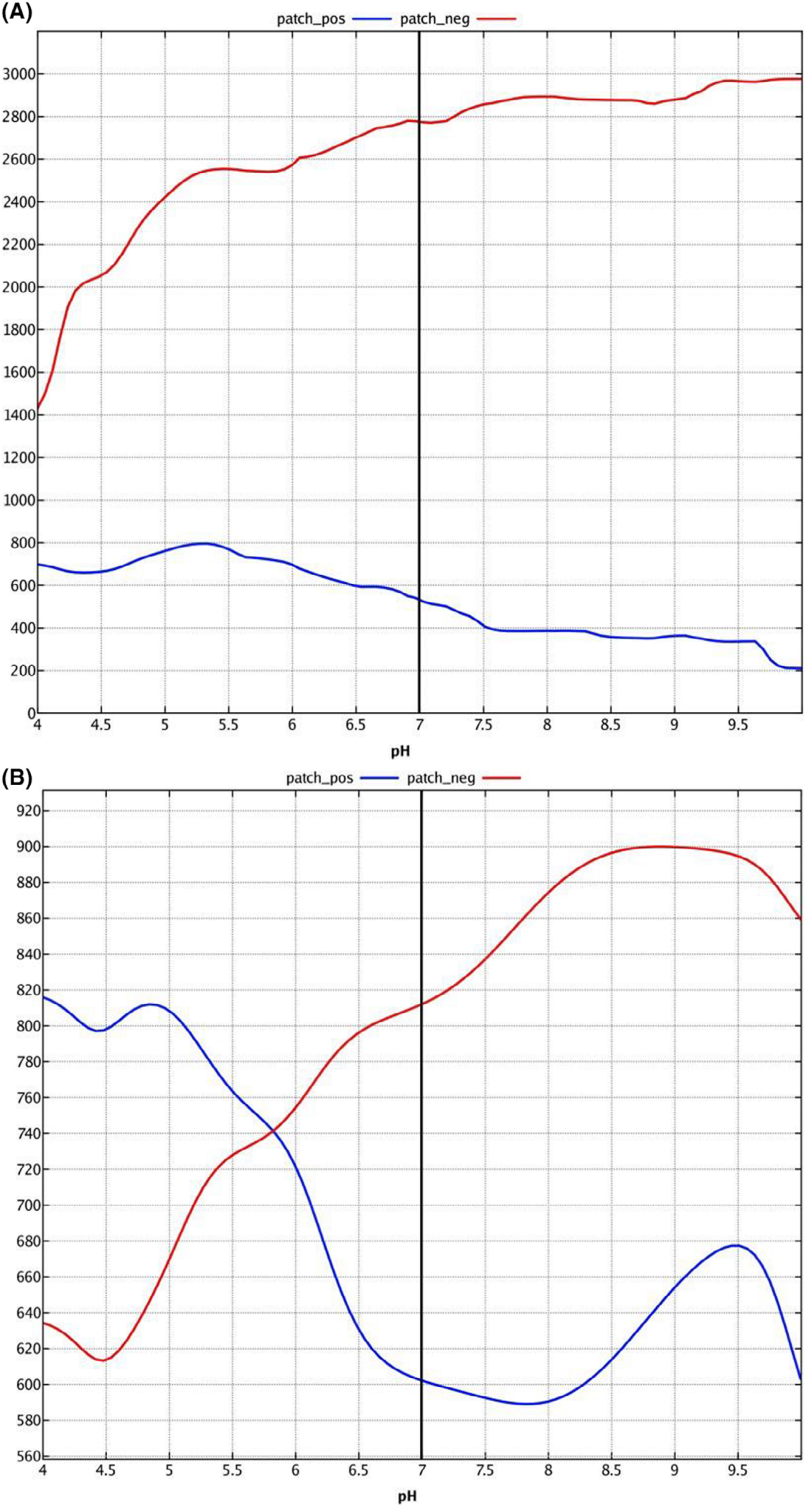
pH‐dependent plots area of negative and positive protein patch (Å^2^). (A) Predictive halophilic endoglucanase from *Stachybotrys microspora*. (B) Predictive nonhalophilic endoglucanase from *S. microspora*.

We also note that the ratio (area of negative/ionic protein patch), which translates the percentage of the negative charge compared with the total charged surface is dominant for the halophilic endoglucanase (84% and 92%) than the nonhalophytic endoglucanase (57%). The analysis of the area of the negative and positive protein patch with a range of pH values from 4 to 10 is shown in Fig. 6. We note that the area of the negative protein patch remains much higher than the area of the positive protein patch for the halophilic endoglucanase when the pH varies from 4 to 10, while we can have an equality of the area of positive and negative for the nonhalophilic endoglucanase when the pH is between 5.5 and 6. These results are in line with the fact that halophilic endoglucanases are more negatively charged.

### Docking of the predicted halophilic endoglucanase with its substrate

Moreover, docking studies can provide important information and are a very useful tool for understanding the prevailing binding modes between proteins and ligands. In our work, we performed blind docking of CT3 (β‐cellotriose, a small substrate used to study endoglucanase activity) using the SwissDock web‐based program in order to investigate the possible modes of interaction. Figure S5 shows that the best binding solutions are located in the same regions with values of FullFitness ranging from −2178.56 to −173.97 kcal·mol^−1^ and with an estimated Δ*G* of −7.2.

## Conclusion

Previous biochemical studies carried out in our laboratory have shown the production of cell wall degrading enzymes from *S. microspora*. In fact, our strain is characterized by growing in a cellulosic‐based medium over a wide pH range of 4–9, a very rare feature reported in most known fungal species. In addition to its ability to grow at alkaline pH, *S. microspora* produces neutral/alkaline and halophilic endoglucanases and secretes several β‐glucosidases. Sequencing the entire genome of this rare fungus is crucial for advancing knowledge of its genomic properties. The genome sequence of *S. microspora* consists of 3715 contigs with a genome size of 35 343 854 bp and a GC content of 53.31%. Two bioinformatic tools, Omicsbox and the fungicompanion web server, were used to identify 46 cellulases, confirming the abundance of cellulolytic enzymes. An endoglucanase, named seq10, was predicted to exhibit a halophilic profile, compared to other annotated and analyzed endoglucanases and was chosen for deeper analyses. It was shown to share strong structural resemblance with a known bacterial halophilic enzyme. These findings corroborate prior biochemical research on *S. microspora* halophilic endoglucanases, which is a very unusual feature for known fungal cellulases. All of these characteristics make *S. microspora* ideal for a wide range of applications, including biofuel, textiles, and detergents.

## Conflict of interest

The authors declare no conflict of interest.

## Author contributions

SA, OV, and AG were responsible for the study design. SA, IB, IBH, and NK performed the experiments. FF and SA performed the structural analysis. SA wrote the first draft of the article. OV, BK, AL, and AG supervised the draft of the article. All of the authors approved the final version of the article submitted for publication.

## Supporting information


**Fig. S1.** A flow diagram of the steps for functional annotation with the OmicsBox platform.

**Fig. S2.** Sample results after functional annotation under OmicsBox platform.

**Fig. S3.** A sample results analysis of family 3 β‐glucosidase under OmicsBox platform.

**Fig. S4.** Phylogenetic tree for some known marine fungi with *S. chartarum* and *S. chlorohalonata* compared to *S. microspora* using MEGA X package.

**Fig. S5.** Visualization of the predicted GH5 endoglucanase from *S. microspora* with different modes of binding using β‐cellotriose (CT3) as a ligand.

## Data Availability

The *S. microspora* genome sequence project has been deposited in the DDBJ/EMBL/GenBank databases under the accession number JAEVLY000000000, the BioProject number PRJNA694831, and the BioSample number SAMN17575027.
