# Supplementary figures and images for: Large‐scale analysis of the genome of the rare alkaline‐halophilic Stachybotrys microspora reveals 46 cellulase genes
Source: FEBS Open Bio. 2023 Feb 17;13(4):670–83. doi: 10.1002/2211-5463.13573 (PMC10068326; doi:10.1002/2211-5463.13573)

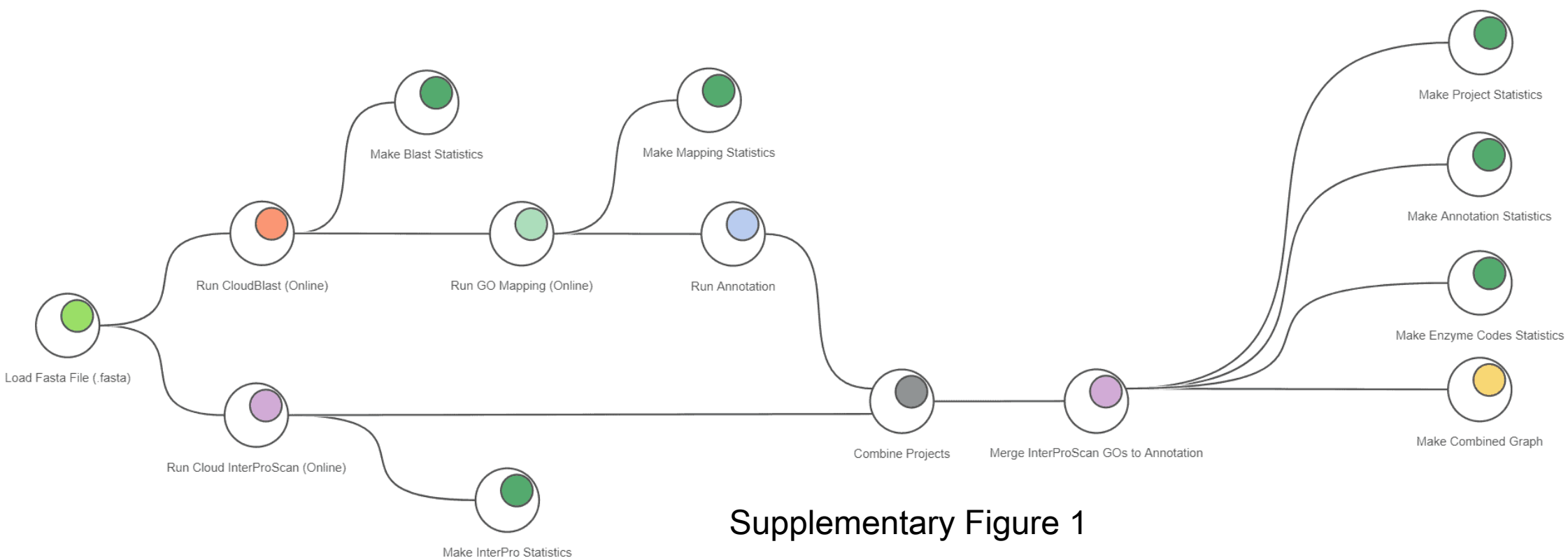

Supplement: Supplementary file 1 — Fig. S1. A flow diagram of the steps for functional annotation with the OmicsBox platform. [file FEB4-13-670-s001.pdf]

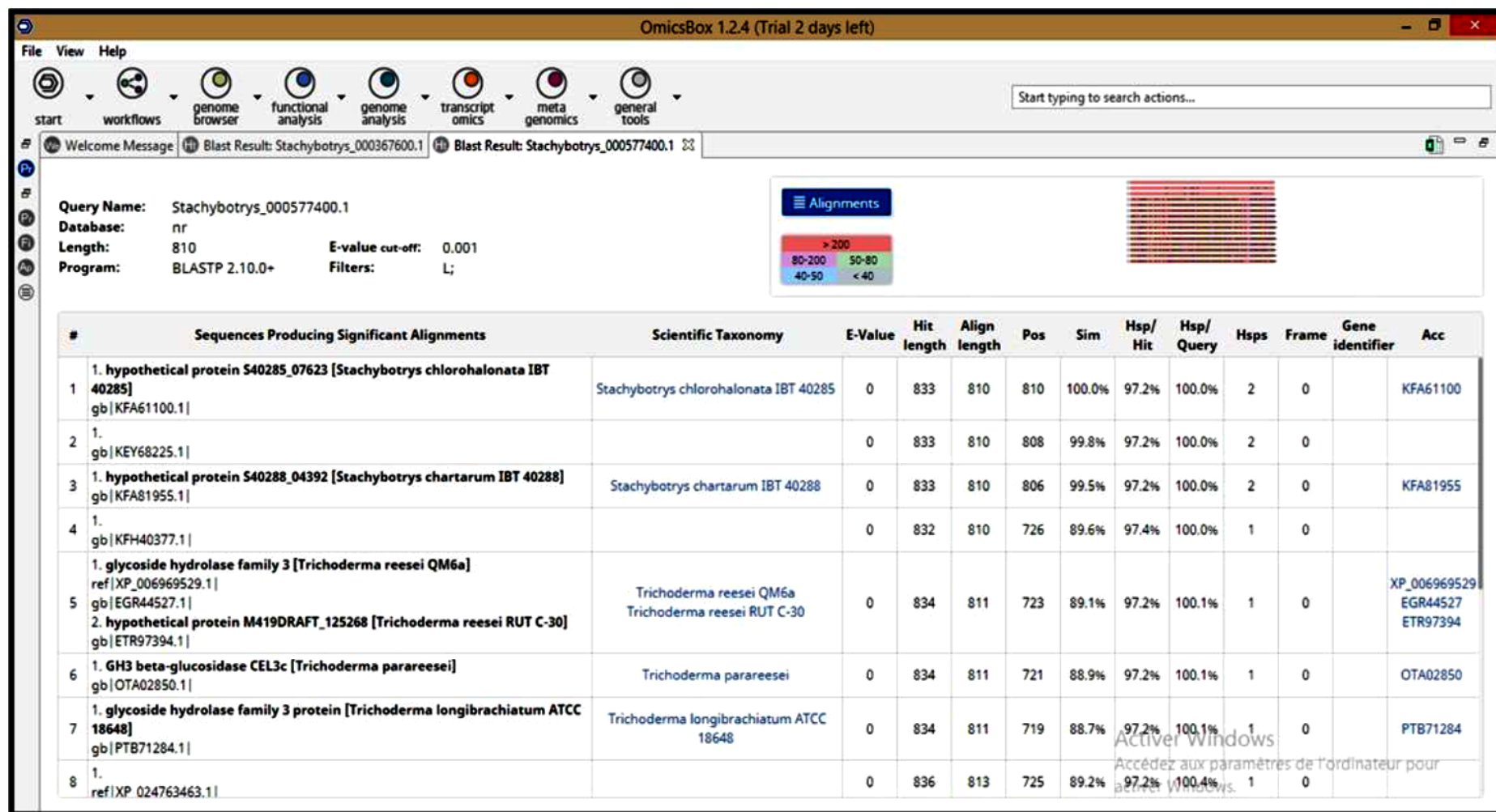

Supplementary Figure 3

Supplement: Supplementary file 3 — Fig. S3. A sample results analysis of family 3 β‐glucosidase under OmicsBox platform. [file FEB4-13-670-s002.pdf]

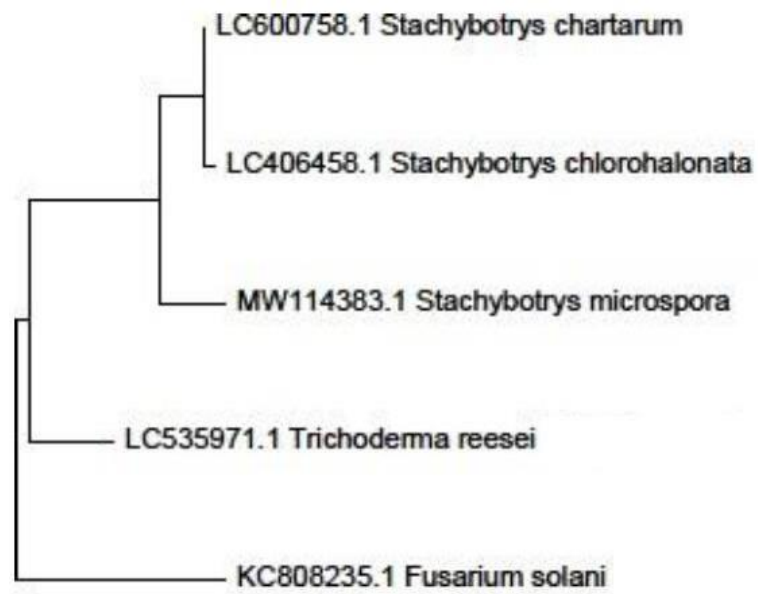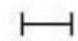

0.020

**Supplementary Figure 4**

Supplement: Supplementary file 4 — Fig. S4. Phylogenetic tree for some known marine fungi with S. chartarum and S. chlorohalonata compared to S. microspora using MEGA X package. [file FEB4-13-670-s004.pdf]

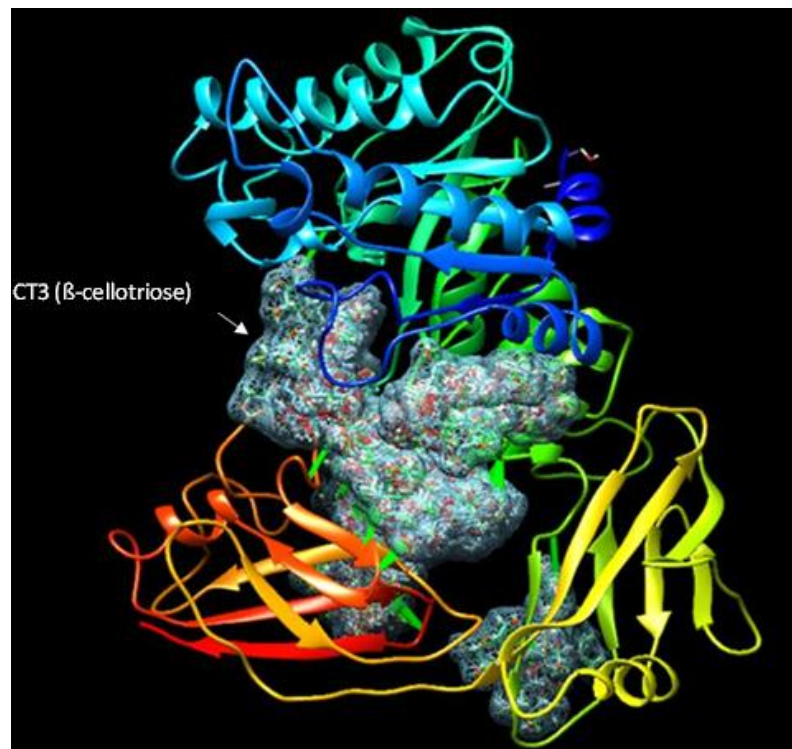

**Supplementary Figure 5**

Supplement: Supplementary file 5 — Fig. S5. Visualization of the predicted GH5 endoglucanase from S. microspora with different modes of binding using β‐cellotriose (CT3) as a ligand. [file FEB4-13-670-s003.pdf]
