# Supplementary material for: Large‐scale analysis of the genome of the rare alkaline‐halophilic Stachybotrys microspora reveals 46 cellulase genes
Source: FEBS Open Bio. 2023 Feb 17;13(4):670–83. doi: 10.1002/2211-5463.13573 (PMC10068326; doi:10.1002/2211-5463.13573)
Supplement: Supplementary file 2 — Fig. S2. Sample results after functional annotation under OmicsBox platform. [file FEB4-13-670-s005.pdf]

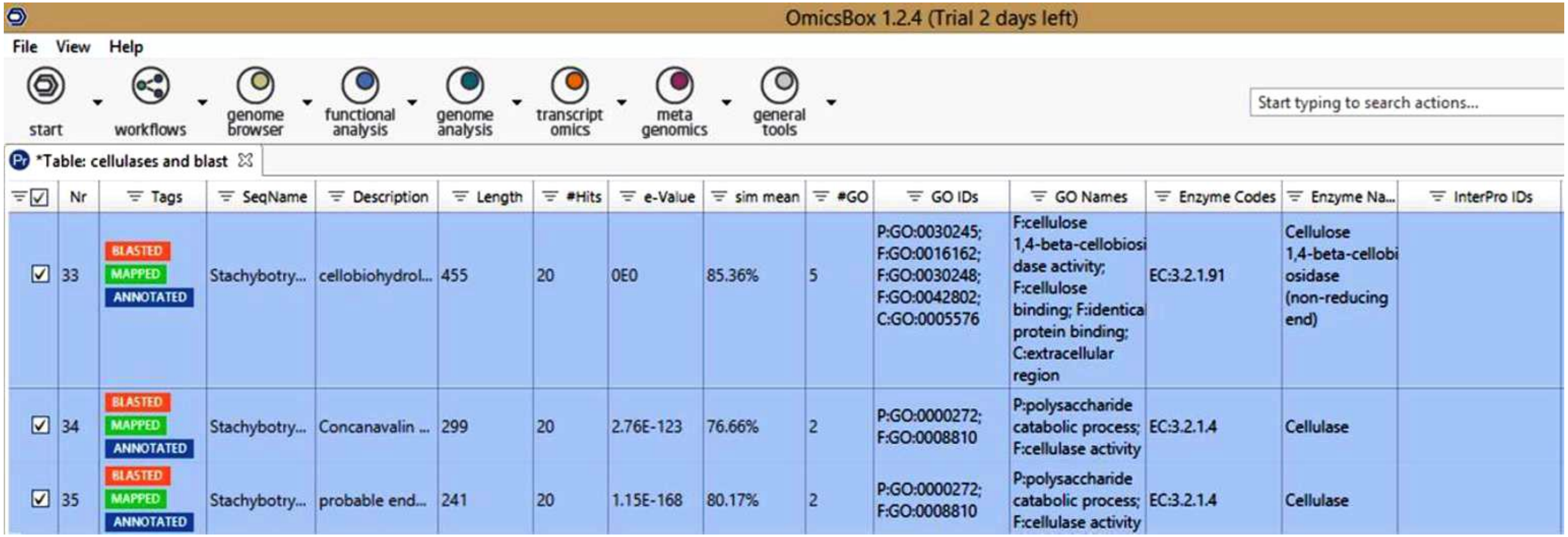

☑	Nr	Tags	SeqName	Description	Length	#Hits	e-Value	sim mean	#GO	GO IDs	GO Names	Enzyme Codes	Enzyme Na...	InterPro IDs
☑	33	BLASTED MAPPED ANNOTATED	Stachybotry...	cellobiohydrol...	455	20	0E0	85.36%	5	P:GO:0030245; F:GO:0016162; F:GO:0030248; F:GO:0042802; C:GO:0005576	F:cellulose 1,4-beta-cellobiosidase activity; F:cellulose binding; F:identica protein binding; C:extracellular region	EC:3.2.1.91	Cellulose 1,4-beta-cellobiosidase (non-reducing end)	
☑	34	BLASTED MAPPED ANNOTATED	Stachybotry...	Concanavalin ...	299	20	2.76E-123	76.66%	2	P:GO:0000272; F:GO:0008810	P:polysaccharide catabolic process; F:cellulase activity	EC:3.2.1.4	Cellulase	
☑	35	BLASTED MAPPED ANNOTATED	Stachybotry...	probable end...	241	20	1.15E-168	80.17%	2	P:GO:0000272; F:GO:0008810	P:polysaccharide catabolic process; F:cellulase activity	EC:3.2.1.4	Cellulase	

Supplementary Figure 2
